# 307. Respiratory Syncytial Virus (RSV) Infections Among Persons Aged 60 Years and Older, by Vaccination Status, reported via Electronic Laboratory Reporting (ELR) – Los Angeles County (LAC), California

**DOI:** 10.1093/ofid/ofae631.097

**Published:** 2025-01-29

**Authors:** Heidi Ransohoff, Natalie A Frey, Olivia Moir, Elizabeth Traub, Andrea Kim, Nava Yeganeh, Annabelle de St. Maurice

**Affiliations:** Los Angeles Department of Public Health, Los Angeles, California; Los Angeles County Department of Public Health, Van Wert, Ohio; Los Angeles County Department of Public Health, Van Wert, Ohio; Los Angeles County Department of Public Health, Van Wert, Ohio; Los Angeles County Department of Public Health, Van Wert, Ohio; LACDPH, Los Angeles, California; Los Angeles County Department of Public Health, Van Wert, Ohio

## Abstract

**Background:**

On June 21, 2023 the CDC Advisory Committee on Immunization Practices recommended 2 different RSV vaccines for adults ≥ 60 years with shared clinical decision making. This study aims to compare the risk of laboratory-confirmed RSV infection in presumed vaccinated and unvaccinated adults ≥ 60 years living in LAC.

**Figure:**
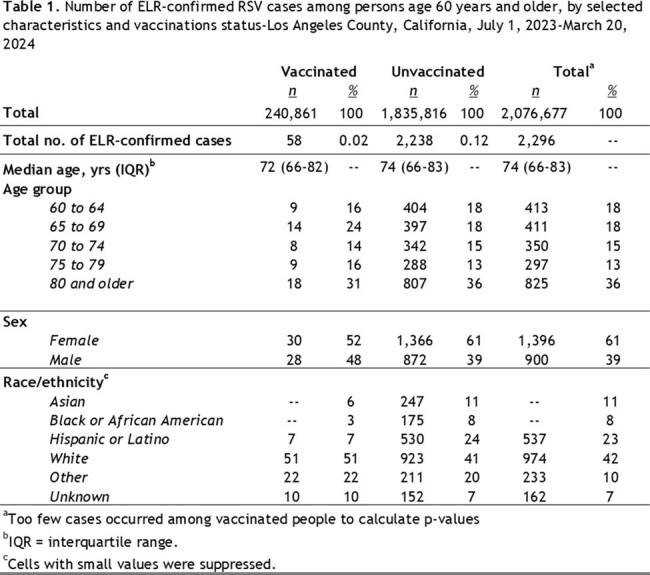

**Methods:**

A population-based analysis was conducted using RSV vaccination data from the California Immunization Registry (CAIR), RSV positive case data reported to LAC Department of Public Health (DPH) via ELR between July 1, 2023 to March 20, 2024, and July 1, 2022 Population Estimates (provisional), prepared by Hedderson Demographic Services for Los Angeles County Internal Services Department, released March 2023. The study population included LAC residents ≥ 60 years at time of specimen collection. The data were matched on name, date of birth, and street address. Vaccinated cases were defined as persons who had CAIR record of RSV vaccination ≥ 14 days before specimen collection.

**Results:**

The population of LAC residents ≥ 60 years is estimated at 2,076,677; 240,861 had RSV vaccinations in CAIR. 2,296 were ELR RSV cases: 58 (2.5%) were vaccinated; 2,238 (97.5%) were unvaccinated. The risk of ELR RSV infection among unvaccinated persons was 5.1 times higher than among vaccinated persons (Table 1; RR= 5.1; 95% CI 3.7:5.1, p < 0.001). Most cases were older than 74.

**Conclusion:**

The results show that unvaccinated older adults in LAC had a higher risk of ELR RSV infection than those who were vaccinated. Limitations include possible incomplete data in CAIR and ELR, making infection risk estimates imprecise. Future studies using these data sources may include hospitalization and death data to compare the incidence of serious illness in vaccinated and unvaccinated adults.

**Disclosures:**

**All Authors**: No reported disclosures

